# Performance and Biases of the LENA and ACLEW Algorithms in Analyzing Language Environments in Down, Fragile X, Angelman Syndromes, and Populations at Elevated Likelihood for Autism

**DOI:** 10.1111/desc.70239

**Published:** 2026-07-08

**Authors:** Marvin Lavechin, Lisa R. Hamrick, Bridgette Kelleher, Amanda Seidl

**Affiliations:** ^1^ Department of Brain & Cognitive Sciences Massachusetts Institute of Technology Cambridge Massachusetts USA; ^2^ Department of Psychology University of South Carolina Columbia South Carolina USA; ^3^ Department of Psychological Sciences Purdue University West Lafayette Indiana USA; ^4^ Department of Communication Sciences & Disorders University of Delaware Newark Delaware USA

**Keywords:** automatic analysis, validation study, Down syndrome, fragile X syndrome, Angelman syndrome, elevated likelihood for autism

## Abstract

Wearable recorders are used in research and clinical practice to collect and measure children's vocalizations and the language environment in which they occur. Recordings generate vast amounts of audio, making manual analysis impractical and requiring automated processing. Two automated algorithms have emerged: the proprietary LENA (Language ENvironment Analysis) and the open‐source ACLEW (Analyzing Child Language Experiences around the World) systems; yet, systematic performance comparisons remain scarce. Here, we validate and compare the performance of these two algorithms across key measures: audio segmentation into speaker categories, conversational turn count (CTC), adult word count (AWC), and child vocalization count (CVC). This analysis is based on 25 h of manually annotated audio recordings from 50 age‐matched U.S. children with diverse neurodevelopmental profiles: children with Down syndrome, Fragile X syndrome, and Angelman syndrome, children at elevated likelihood of autism, and low‐risk controls. We hypothesized that the algorithms might be less accurate for children with neurodevelopmental conditions, since these children often show different patterns of volubility and vocal maturity compared to the typically developing children used to train the algorithms. Thus, we assessed the performance of algorithms across diagnostic groups, a crucial validation step for both cross‐population research and the evaluation of language interventions. Results reveal that while algorithms achieve similar performance across groups, they show different patterns: LENA makes fewer segmentation mistakes but misses many segments (identification error rate = 81.3%, percent correct = 45.3%), while ACLEW shows the opposite pattern (identification error rate = 129.4%, percent correct = 69.4%). Both LENA and ACLEW achieve reasonable levels of accuracy in their automatic counts (Pearson's *r* ranging from 0.78 to 0.92) and maintain stable performance across diagnostic groups. We conclude with recommendations for the validation and potential use of these algorithms in research and clinical practice.

**Summary:**

- Our comparison of the LENA and ACLEW algorithms in analyzing children's language environment and vocal production across five neurodevelopmental profiles reveals similar performance but distinct error patterns.
- LENA makes fewer errors but misses speech (81.3% error rate, 45.3% correct); ACLEW makes more errors but captures more speech (129.4% error rate, 69.4% correct).
- Both algorithms maintain consistent performance across diagnostic groups, supporting their reliability for research with these 2‐year‐old populations with diverse neurodevelopmental profiles.
- Variations in algorithm performance are primarily driven by the total speaking time of surrounding speakers (other children and adults), rather than the diagnostic group.

## 1 Introduction

Children with neurogenetic syndromes often display distinctive patterns of volubility and vocal development compared to typically developing children, making the monitoring of their vocal production and language environment particularly valuable for guiding interventions and informing prognoses (Belardi et al. 2017; Berger and Cunningham 1983; Lohmander et al. 2017; Marschik et al. 2014). However, monitoring children's vocal production and language environment is challenging for numerous reasons. First, vocal behaviors in infants are both variable and context‐dependent, making consistent measurement difficult even in typically developing populations (Soderstrom and Wittebolle 2013). This variability is further amplified in children with neurogenetic syndromes, particularly those with minimal vocalization skills who may produce vocalizations too infrequently to establish reliable baselines (Grieco et al. 2018). Because in‐lab assessments are inherently noisy, and sampling occurs infrequently, small but meaningful changes in vocal production are often obscured. Second, the rarity of neurogenetic conditions (e.g., 1 in 20,000 for Angelman syndrome; Dagli et al. 2021) creates logistical challenges. Families must often travel long distances to specialized research centers, creating a substantial burden and disproportionately affecting families whose children exhibit severe behavioral difficulties or those with limited financial resources (e.g., inability to take paid time off).

**FIGURE 1:**
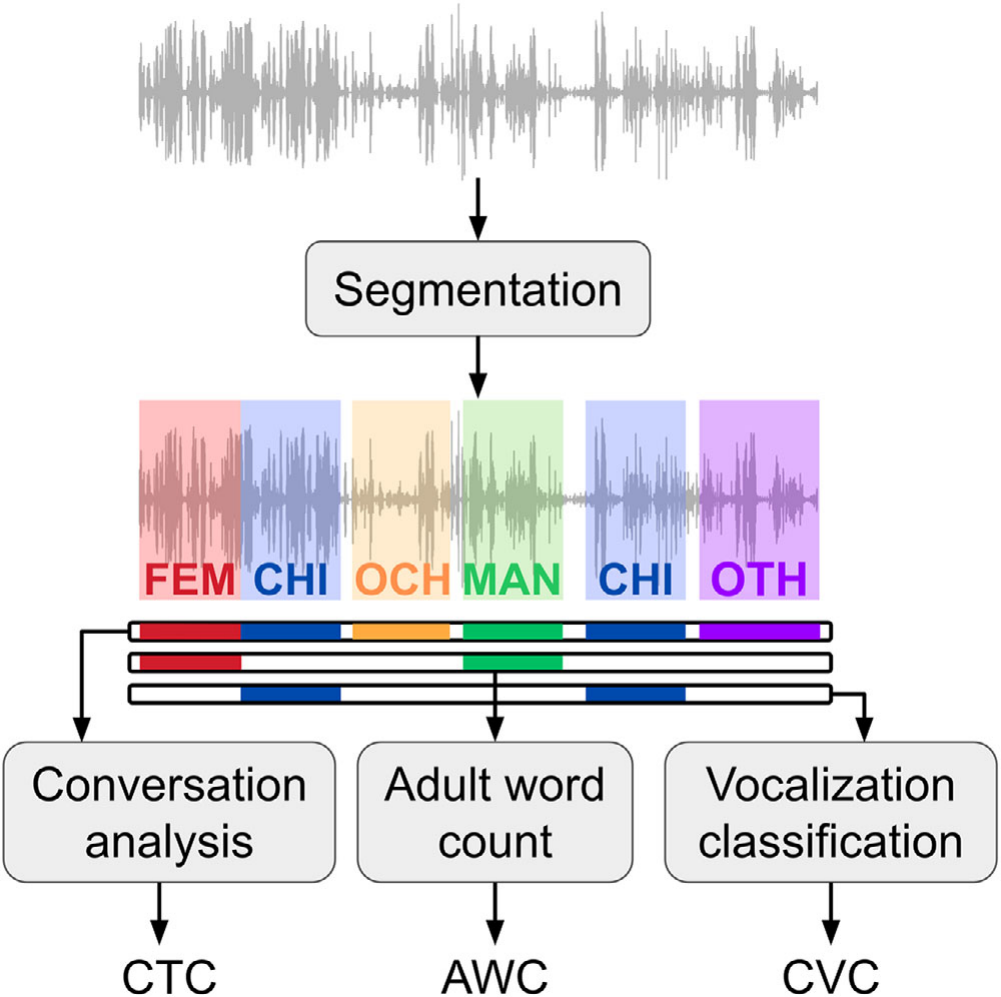
Overview of the LENA and ACLEW systems for analyzing child‐centered daylong recordings. After a segmentation step where key sound events are detected, subsequent steps extract various measures of the child's language environment and production. Detected categories are shown as FEM (female adult), CHI (key child), OCH (other children), MAN (male adult), and OTH (other categories). Typical measures include CTC (conversational turn count) computed as the number of times the key child vocalizes and an adult follows (or vice versa) within a 5‐s window, AWC (adult word count) computed as the number of words produced by adult speakers, CVC (child vocalization count) computed as the number of speech‐like vocalizations produced by the key child.

Lightweight child‐worn recorders offer an appealing solution to these challenges, as the technology can be shipped for remote data collection, and the recorders themselves are unobtrusive and well‐received by most child populations—see Casillas and Cristia (2019) for an introduction to the technique and Cychosz et al. (2020) for ethical considerations. By capturing the child's language environment and vocal production, these daylong recordings (also known as long‐form recordings) provide a uniquely naturalistic window into the child's spontaneous vocalizations and communicative exchanges during daily routines and social activities. The LENA system is a prominent example of this technology, specifically designed for studying early language development (Gilkerson et al. 2008; Gilkerson and Richards 2008a; Xu et al. 2008). It consists of a small digital recorder worn in a specifically designed pocket on the child's clothing that records audio for up to 16 h (*the hardware*), paired with a proprietary algorithm that automatically analyzes the audio data (*the software*). After detecting key sound events occurring in the audio, the LENA algorithm extracts estimates of children's and caregivers’ speech, including how many conversational turns occur between the key child and an adult (CTC; see Table 1 for a list of acronyms), how many words adults say around the child (AWC), and how many speech‐like sounds the child makes (CVC)—see Figure 1 for an overview of the algorithm. Since its development in the early 2000s, LENA has been widely adopted in theoretical and clinical research, across languages and neurodevelopmental profiles (e.g., Oller et al. 2010; Ganek and Eriks‐Brophy 2018a, b; McDonald et al. 2021; Cristia et al. 2021; see also Putnam et al. 2024 for a recent review on the use of LENA for autism research).

**TABLE 1:** List of acronyms used throughout this study.

Acronym	Full name	Type
LENA	Language ENvironment Analysis	Algorithm
ACLEW	Analyzing Child Language Experiences around the World	Algorithm
VTC	Voice Type Classifier	ACLEW component
ALICE	Automatic LInguistic unit Count Estimator	ACLEW component
VCMNet	VoCal Maturity Net	ACLEW component
CTC	Conversational turn count	Measure
AWC	Adult word count	Measure
CVC	Child vocalization count	Measure
LR	Low risk	Group
DS	Down syndrome	Group
FXS	Fragile X syndrome	Group
AS	Angelman syndrome	Group
Asib	Children at elevated likelihood for autism (siblings of children with autism)	Group

Recent technological advancements have dramatically reduced the cost and increased the accessibility of wearables (Islam et al. 2024). As affordable hardware options have multiplied, researchers have sought free, open‐source, and more flexible machine‐learning solutions to analyze these recordings, leading to the development of the ACLEW (Analyzing Child Language Experiences around the World) algorithm. Unlike the LENA algorithm, which is only usable with LENA recorded audios, the ACLEW algorithm can be used with recordings from any device, allowing researchers and practitioners to use the device that best fits their goals. Despite these advantages, the ACLEW algorithm has not undergone the same extensive validation as the LENA system (see previous validation studies). As a result, it remains unclear how the ACLEW algorithm compares to the LENA algorithm.

Beyond comparing the overall performance of these two algorithms, a critical consideration for researchers studying neurodevelopmentally diverse populations is the potential for *algorithmic biases*, that is, systematic performance disparities that affect certain demographic groups. Such biases could have serious clinical implications: If an algorithm systematically underestimates child vocalizations in certain populations, clinicians might incorrectly conclude that these children need more intensive speech therapy, while overestimation could lead to missed opportunities for early intervention. Similarly, biased turn count estimates could result in misguided recommendations about parent–child interactions.

The risk of such biases is particularly concerning given that speech processing algorithms are trained on datasets that may not accurately represent the full range of vocal characteristics in children with neurodevelopmental conditions. Children with Down syndrome and Fragile X syndrome often produce fewer vocalizations and show delays in canonical babbling compared to their typically developing peers (Berger and Cunningham 1983; Lynch et al. 1995; Lohmander et al. 2017; Belardi et al. 2017). Higher pitch and unusual prosodic patterns, often associated with autism (Fusaroli et al. 2022), are more common in neurogenetic syndromes due to the higher prevalence of autism in these populations. For example, these populations can display anywhere from double the prevalence of autism versus low‐risk controls (e.g., see DiGuiseppi et al. 2010 in Down syndrome) to more than 10 times the prevalence (e.g., see Kaufmann et al. 2017 in males with Fragile X syndrome). Distinct facial morphology associated with neurogenetic conditions may further affect vocal production (e.g., see Bull 2020 for populations with Down syndrome). These characteristics can potentially lead algorithms to make more errors on certain populations than others (e.g., missing brief speech segments entirely, incorrectly attributing child vocalizations to adults due to unexpected prosodic patterns, or misclassifying speech‐like sounds as nonspeech‐like due to unfamiliar acoustic properties). These concerns are particularly relevant for the LENA and ACLEW algorithms, as both were developed and optimized using recordings from children with typical language development or no known risks of language delay (Gilkerson and Richards 2008b; Al Futaisi et al. 2019; Lavechin et al. 2020; Räsänen et al. 2021).

**Previous validation studies**. The LENA algorithm has been extensively validated across languages and neurodevelopmental profiles (e.g., see Canault et al. 2016 for French; McDonald et al. 2021 for Korean; Jones et al. 2019 for populations with autism; Bredin‐Oja et al. 2018 for one of the few validation studies across diverse neurodevelopmental profiles). A systematic review by Cristia et al. (2020) analyzing 28 articles revealed that LENA's segmentation algorithm performs reasonably well, with 68% precision and 59% recall. LENA's adult word count (AWC) and child vocalization count (CVC) showed strong correlations with human counts (Pearson's *r* of 0.79 and 0.77, respectively). However, a more problematic level of performance was found for CTC with a Pearson's *r* of only 0.36. Among these 28 articles, seven included clinical populations (autism, elevated autism likelihood, language delays, or preterm infants), demonstrating interest in automatic solutions for assessing and supporting these groups. Notably, these validation studies vary in their methodology: Some compare LENA's output to manual annotation on short clips (which may fail to capture infrequent vocalizers) while others use longer clips (which may fail to sample the day's full variability).

The performance of the ACLEW algorithm remains less thoroughly documented, with different components of the algorithm validated separately across various publications (Al Futaisi et al. 2019; Lavechin et al. 2020; Räsänen et al. 2021). Lavechin et al. (2020) report slightly higher performance in terms of F‐score for ACLEW's segmentation into speaker categories compared to LENA (40.8% for LENA vs. 52.0% for ACLEW when collapsing across speaker categories)—see Long et al. (2024) or Sun et al. (2024) for independent validation studies on non‐LENA‐recorded audios. Among the three automatic counts provided by ACLEW, only AWC has been validated, showing performance comparable to LENA (Räsänen et al. 2021). To the best of our knowledge, the other two counts, CTC and CVC, have not been validated under real‐world conditions (i.e., using automatic rather than human segmentation). Notably, none of the studies mentioned in this paragraph included children with neurogenetic syndromes.

Regarding algorithmic biases, research remains notably scarce despite its fundamental importance for ensuring valid scientific conclusions that are not artifacts of algorithmic performance variations. Seidl et al. (2018) verified the accuracy of LENA's speaker segmentation in siblings of children with autism and found performance comparable to previously reported data for typically developing children; however, this comparison relied on descriptive observation rather than formal statistical analysis. Similarly, Rankine et al. (2017) focus on examining bias within the clinical population (testing factors such as age) rather than testing for bias between typically developing and clinical populations. Similar approaches are used across other populations, such as low‐ versus high‐income families (e.g., Oetting et al. 2009). One exception to this is Cristia et al. (2021), which tested for significant performance differences across different linguistic and socio‐cultural settings.

**The present study**. In this work, we conduct the first systematic comparison of the LENA and ACLEW algorithms using recordings gathered from children with diverse neurodevelopmental profiles. Our research addresses two critical questions as follows: (1) How do these algorithms compare in their ability to segment audio into distinct speaker categories (key child, other children, male adult, female adult) and extract language environment measures (CTC, AWC, CVC) in naturalistic recordings? and (2) Do these algorithms exhibit systematic biases across diagnostic groups (low‐risk controls, Down syndrome, Fragile X syndrome, Angelman syndrome, and siblings of children with autism)? To address these questions, we first compare algorithm performance using our full sample of children with diverse neurodevelopmental profiles in Section 3.1, examine whether performance varies across specific diagnostic groups in Section 3.2, and explore other predictors of performance in Section 3.3.

We evaluate algorithm performance by randomly sampling fifteen 2‐min segments from daylong recordings of 50 age‐matched children (10 per diagnostic group), which were then manually annotated by a single human expert to establish a gold standard. This approach allows us to quantify the performance of both the LENA and ACLEW algorithms across their key measures.

By validating these algorithms on neurodevelopmentally diverse populations, we hope our results will inform researchers and clinicians on which algorithm best fits their needs, and guide future algorithm development to better serve children across the developmental spectrum—particularly children with neurodevelopmental conditions who may benefit most from these tools yet pose unique challenges for algorithm accuracy due to their distinct language patterns.

## 2 Methods

### 2.1 Corpora

#### 2.1.1 Participants

The daylong recordings used for evaluation were subsampled from the Neurodevelopmental Natural History Study, aimed at capturing early development across diverse neurodevelopmental profiles (Kelleher et al. 2020; Hamrick et al. 2023). Across the study, more than 300 families were recruited from various locations in the United States, and a subset of 229 participants (49% were female, mean age 26 months ± 15 months, range 3–81 months) completed at least one full day of recording using LENA recorders (Gilkerson and Richards 2008a).

#### 2.1.2 Selection Procedure

Of the 229 children with LENA data, we used the selection procedure described below to select 50 participants across five diagnostic groups (10 children per group): low‐risk controls (children with no known language delays or risk of language delays), children with Angelman syndrome, Fragile X syndrome, or Down syndrome, and children at elevated likelihood for autism (siblings of children with autism). All children were from American English–speaking families. Children were selected to be approximately 20–22 months old, as this was the only age range for which we could identify a sufficient number of age‐matched children across all five diagnostic groups within our dataset.

We matched children based on chronological age rather than mental age for two reasons. First, matching on mental age would require selecting older children with neurodevelopmental conditions (or younger low‐risk children), introducing *age‐related* acoustic confounds in vocal tract length and pitch that are unrelated to the neurodevelopmental conditions themselves (Fitch and Giedd 1999). Second, it would minimize *condition‐related* vocal differences between groups (e.g., lower volubility or reduced consonant‐vowel combinations), preventing us from testing whether these characteristics affect algorithm performance (Berger and Cunningham 1983; Belardi et al. 2017; Lohmander et al. 2017). Specifically, we selected chronological age‐matched children from each diagnostic group using an iterative matching procedure. For each iteration (*n_iterations_* = 1000), we randomly selected 10 children from a reference group and used the Hungarian algorithm (Kuhn 1955) to find the best age‐matched children from other groups, minimizing age differences between pairs. We kept the selection with the smallest maximum difference in mean age between any two groups. Demographics of our participants are reported in Table 2.

**TABLE 2:** Demographic characteristics of the 50 children included in our sample.

	Low	Angelman	Fragile X	Down	Sibling with
	risk	syndrome	syndrome	syndrome	autism
Sex (M/F)	6/4	4/6	7/3	6/4	3/7
Race					
White	7	7	9	9	8
Black/African American	0	0	0	0	1
Asian	0	1	0	1	0
Not reported	3	2	1	0	1
Ethnicity					
Not Hispanic or Latino	8	7	9	9	8
Hispanic or Latino	0	1	0	1	2
Not reported	2	2	1	0	0
Mother highest level of education					
High school or less	1	0	1	0	1
Some college	0	2	2	1	5
Bachelor's degree	2	2	2	3	2
Advanced degree	7	4	5	6	0
Not reported	0	2	0	0	2
Father highest level of education					
High school or less	0	0	2	1	1
Some college	2	2	2	2	5
Bachelor's degree	4	3	4	3	2
Advanced degree	2	3	2	4	0
Not reported	2	2	0	0	2
Total household income					
<$70k	5	0	1	1	3
$70k–$120k	4	3	1	3	3
$120k–$200k	1	1	4	2	2
>$200k+	0	3	3	1	0
Not reported	0	3	1	3	2

***Annotation procedure***. Following Cristia et al. (2021), we randomly sampled fifteen 2‐min nonoverlapping clips from each daylong recording (one daylong recording per child). This sampling strategy maximizes the diversity of captured interactions while maintaining feasible annotation time constraints since a larger number of shorter clips are more likely to capture distinct interaction types than fewer, longer clips. Another sampling strategy often used in the literature is to select high‐volubility clips based on automatic annotation (Soderstrom et al. 2021), but this would have introduced a selection bias into our study given the populations explored. Besides, by selecting segments already identified as speech‐dense by either system, we would over‐represent interactions that the respective system detects, artificially inflating its performance metrics. This sampling procedure yielded 25 h of audio.

A single human expert annotated all clips following the DARCLE annotation scheme (Casillas et al. 2017), marking onsets and offsets of all speech and vocalizations and assigning each segment to speaker categories (key child, other children, female adults, male adults). Key child vocalizations were classified by type (canonical, noncanonical, crying, laughing) to derive CVC, and adult speech was transcribed to derive AWC. In total, 5.5 h of speech/vocalizations (from children and adults) were identified and annotated.

Our human expert is a native American English speaker with a background in vocal development and many years of experience annotating infant speech data. They were masked to the participants' diagnostic group status during tagging. While we acknowledge that multiple annotators would be ideal, we have little reason to be concerned about annotation reliability in this context, as the DARCLE annotation scheme has been previously validated on LENA daylong recordings with a Cohen's *κ* of 0.64 (Cristia et al. 2021), though we note that this validation was conducted on typically developing populations. The composition of our annotated dataset is reported in Table 3.

**TABLE 3:** Sample characteristics of participants and clips used for evaluating the LENA and ACLEW systems.

Group	Number of children	Number of 2‐min clips	Age range (mean ± sd)
Low risk	10	150	22.0 ± 3.9
Angelman syndrome	10	150	21.0 ± 4.8
Fragile X syndrome	10	150	22.0 ± 4.5
Down syndrome	10	150	21.5 ± 3.5
Sibling with autism	10	150	20.5 ± 4.9

### 2.2 Algorithms

First, we describe how LENA and ACLEW analyze children's language environments and production at a high level. We then provide a short technical overview of both systems before presenting their key differences and how we handled them to allow for comparison.

#### 2.2.1 High‐Level Overview

From a high‐level perspective, the LENA and ACLEW systems work similarly (see Figure 1).

Upon receiving an audio recording, both systems start by segmenting it into different categories. The key categories for the present work are CHI, which indicates when the system predicts a vocalization from the key child (the child wearing the recording device), OCH for vocalizations from any other children, FEM for female adult speech, and MAL for male adult speech. This segmentation serves as input for three downstream analyses. Conversation analysis (computing turn‐taking between the key child and adults), from which the CTC measure is derived; adult word count (estimating the number of words produced by adult speakers), from which the AWC measure is derived; and vocalization classification (classifying child segments as speech‐like or nonspeech‐like), from which the CVC measure is derived.

#### 2.2.2 Technical Overview and Key Differences Between LENA and Aclew

Despite similar approaches, the LENA and ACLEW systems have important differences. Here, we discuss some of these differences and, when relevant, explain how we handled them to allow for comparison between the two systems.

***Segmentation and conversation analysis***. LENA segments the audio into speaker and nonspeaker categories using a minimum duration Gaussian mixture model (MDGMM) fed with 36 mel‐frequency cepstrum coefficients (Xu et al. 2008). Categories include the following: female adult speech, male adult speech, key child vocalizations, other child vocalizations (all of which will be considered in this paper), as well as electronic speech, overlapping speech, noise, and silence, which are outside the scope of our current evaluation (see Cristia et al. 2021 for an assessment of these additional categories). Additionally, LENA assigns confidence levels to each category: “near” (high probability) and “far” (low probability). Since far categories have been shown to be unreliable (Cristia et al. 2021), our analysis will focus on LENA's “near” speech categories, with all other categories mapped to silence to enable direct comparison with ACLEW (which does not produce nonspeech categories).

In ACLEW, the segmentation step is performed by the Voice Type Classifier (VTC), a deep‐learning model that combines SincNet filters for learning low‐level acoustic representations with long short‐term memory (LSTM) and feed‐forward layers for aggregating these into context‐dependent representations (Lavechin et al. 2020). VTC detects the same speaker categories as LENA (CHI, OCH, FEM, MAL) plus a general SPEECH category that encompasses any speech sound regardless of speaker (not relevant for the present study).

Note that the CTC measure is directly computed from this segmentation step. Following LENA's original definition, we calculate CTC for both systems as the number of times the key child vocalizes, and an adult follows (or vice versa) within a 5‐s window, though researchers could compute CTC using different window durations if desired.

***AWC***. In LENA, adult speech segments identified in the segmentation step are further processed to estimate word counts using a least‐squares linear regression based on the sequence length and the number of consonants and vowels. These phonetic features are obtained using the Sphinx phone recognizer optimized for American English adult speech (Lamere et al. 2003; Xu et al. 2008).

ACLEW uses ALICE (Automatic LInguistic unit Count Estimator) to estimate AWC (Räsänen et al. 2021). ALICE also consists of a least‐squares linear regression model, but contrary to LENA, it is fed with (1) the estimated number of consonants, vowels, and consonant–vowel or vowel–consonant alternations using the pretrained multilingual phone recognizer Allosaurus (Li et al. 2020); (2) the estimated number of syllables using a pretrained syllable count estimator SylNet (Seshadri and Rasanen 2019); and (3) various signal‐level features such as the utterance duration or the total signal energy.

In both systems, AWC is derived by summing the word count estimates from all adult speech segments detected during the recording.

***Vocalization classification***. In LENA, segments identified as produced by the key child are further classified into vegetative sounds (e.g., breathing or burping), fixed signals (e.g., crying, screaming, laughing), and speech‐like vocalizations. We found little information about how this classification is performed, but Xu et al. (2008) suggest an approach based on low‐level acoustic features discriminative of the type of sounds produced by the child.

In ACLEW, the key‐child segments are further processed by VCMNet (VoCal Maturity Net), a deep‐learning model that classifies vocalizations into canonical (syllables with vowel and consonant sounds), noncanonical (vowel‐only sounds), crying, and other (the latter of which includes laughing and other vegetative sounds such as burping). See Al Futaisi et al. (2019) for implementation details. While our past investigations have revealed that the algorithm shows limited accuracy in distinguishing canonical from noncanonical vocalizations, this limitation is not relevant for the present study, as we merge VCMNet's canonical and noncanonical categories into a single category to match LENA's speech‐like category.

In both systems, CVC is derived by counting the number of speech‐like vocalizations produced by the key child.

***Training data***. The systems differ in their training data. LENA was trained and validated using recordings from monolingual English‐speaking families^1^ (Gilkerson et al. 2008). ACLEW, however, incorporated data from multiple languages, including data from non‐WEIRD (Western, Educated, Industrialized, Rich, and Democratic) communities. Importantly, neither LENA nor ACLEW was trained or validated using recordings from children with known neurodevelopmental conditions or atypical language development.

***User accessibility***. From the user's standpoint, perhaps one of the major differences between LENA and ACLEW is that of accessibility. LENA consists of the recording device and a fully integrated speech‐processing pipeline, allowing users to collect and analyze data seamlessly within a user‐friendly interface that does not require programming skills. The ACLEW system is accessible through GitHub repositories (VTC^2^, ALICE^3^, and VCMNet^4^). Although the documentation assumes minimal programming skills, users still need to download the source code, install dependencies, and run command‐line programs to analyze their recordings. Additionally, access to a computing cluster is necessary if a large volume of data must be analyzed, as the deep learning models (particularly VTC) are computationally intensive and may require substantial processing time on standard computers. That said, and contrary to LENA, ACLEW has the advantage of being free and open‐source, which means that users can run analyses on any recordings, not just those collected using the LENA recorder. Users can therefore employ less expensive recorders, some of which may even be smaller and more portable than the LENA recorder.

### 2.3 Evaluation Metrics

#### 2.3.1 Speaker Segmentation Evaluation

Automatic and manual annotations were standardized into .csv files containing segment onset, duration, and speaker information using ChildProject (Gautheron et al. 2023). These files were used to extract performance metrics of LENA and ACLEW speaker segmentation using ChildProject and pyannote.metrics (Bredin 2017). The following section details our performance metrics.

***Identification error rate and percentage correct***. Two performance metrics often used in tandem for evaluating segmentation algorithms are the identification error rate and percentage correct. At any point in the recording, one of the following four scenarios occurs: (1) the algorithm detects speech when there is none (false alarm), (2) the algorithm detects nothing when there is speech (miss), (3) both the algorithm and the annotator detect speech but disagree on the speaker category (confusion), or (4) both detect speech and agree on the category (correct instances). Misses, false alarms, and confusions reveal the algorithm's error patterns and are combined to calculate the identification error rate:

$$\mathrm{identification}\;\mathrm{error}\;\mathrm{rate}=\frac{\mathrm{false}\;\mathrm{alarm}+\mathrm{miss}+\mathrm{confusion}}{\mathrm{total}}$$

To illustrate how these duration‐based metrics capture onset and offset accuracy: If an algorithm predicts a child's vocalization from 10 to 15 s but the human annotator marked it from 12 to 18 s, this creates both 2 s of false alarm (10–12 s, where the algorithm detected speech before it actually began) and 3 s of miss (15–18 s, where the algorithm stopped detecting speech before it actually ended).

Complementary to the identification error rate is the percentage correct, computed as the proportion of times when the algorithm made the right decision:

$$\mathrm{percentage}\;\mathrm{correct}=\frac{\mathrm{correct}}{\mathrm{total}}$$

In both formulas, *total* is the cumulated duration of speech/vocalizations found by the human annotator in the recordings. Since identification error rate and percentage correct are computed relative to speech duration, they do not sum to 100%. While the percentage correct is bounded between 0% and 100%, the identification error rate can exceed 100% due to false alarms occurring during periods of nonspeech.

***Confusion matrix***. A confusion matrix shows how instances are distributed between predicted and actual categories. Each cell (*i*,*j*) is computed as the duration where the automatic system labeled as speaker *i* and the human annotator labeled as speaker *j*. Row normalization (dividing by total system‐predicted duration) reveals true category distribution across predictions, while column normalization (dividing by total human‐labeled duration per speaker) shows prediction distribution across true categories. Row normalization relates to precision (what % of a predicted speaker category was actually correct), while column normalization relates to recall (what % of a true speaker category was correctly identified). The matrix helps visualize error patterns, with off‐diagonal elements revealing confusion between specific speakers.

#### 2.3.2 Estimated Counts Evaluation

For both automatic and manual annotations, the CTC measure was computed as the number of times the key child vocalizes, and an adult vocalization follows (or vice versa) within a 5‐s window. For manual annotations and those returned by the ACLEW system, CVC was counted as the number of times the key child produces canonical and noncanonical vocalizations. LENA's raw CVC estimate, computed as the number of speech‐like vocalizations produced by the key child, was used. For AWC, LENA and ACLEW's raw estimates were used. Human AWC was computed by counting words in adult segments, including unintelligible words marked with x's and separated by spaces from other words.

Here, we introduce the metrics used to evaluate LENA and ACLEW estimated counts (CTC, AWC, and CVC). Unlike the segmentation metrics in Section 2.3.1, these count‐based metrics evaluate only total quantities, not temporal alignment (i.e., an algorithm could achieve low count error even if events are detected at different timepoints, as long as the totals match).

***Error and percentage error***. Error is defined as the difference between the estimated and true counts:

error=estimatedcount−truecount

Percentage error expresses this difference relative to the true count:

$$\mathrm{percentage}\;\mathrm{error}\;=100\;\times \;\frac{\mathrm{correct}}{\mathrm{true}\mathrm{count}}$$

Both metrics indicate estimation accuracy, with values closer to zero suggesting better performance. While error provides the difference in counts, percentage error normalizes this difference by the true count. For instance, consider a system that overestimates AWC by 100 words. When the true count is 500 words, this represents a 20% error. When the true count is 5000 words, the same 100‐word error is only a 2% error. This normalized view helps compare accuracy across different count magnitudes and allows performance to be compared across measurement types (like CTC vs. AWC). For both metrics, negative values indicate underestimation, while positive values indicate overestimation by the automatic system.

***Pearson's r correlation coefficient***. Estimated counts are often used in correlational analysis, for example, correlating the number of adult words (AWC) with the number of speech‐like vocalizations produced by the key child (CVC). For this reason, it may be more relevant to look at error patterns in terms of Pearson's *r* correlation coefficient, defined as:

$${\mathrm{Pearson}}^{\prime }s\;r=\frac{cov(x,y)}{\sigma_{x}\sigma_{y}}$$

where *cov*(*x*,*y*) is the covariance between true and estimated counts, and *σ_x_* and *σ_y_* are their standard deviations. Pearson's *r* ranges from −1 to 1, with 1 indicating perfect positive correlation, 0 indicating no linear relationship, and −1 indicating perfect negative correlation.

#### 2.3.3 Combining 2‐Min Clips Into 30‐Min Megaclips

Instead of computing performance metrics on our 2‐min clips, which may poorly reflect the algorithms' performance on longer time scales, we concatenate our 15 randomly sampled 2‐min clips into 30‐min megaclips. Critically, we concatenate clips first and then compute performance metrics once on the resulting 30‐min megaclip, rather than computing metrics on each 2‐min clip and averaging them. This distinction is important because most performance metrics are normalized by the total duration of speech in the recording (e.g., percentage correct = correct/total). With short clips, the denominator can be extremely small, making metrics volatile: A single error has a disproportionate impact. Concatenating clips increases the denominator, stabilizing metrics and allowing local overestimations and underestimations to offset each other as they would in real‐world analysis of extended recordings (see Figure S1 and Discussion). Note that both algorithms process the full daylong recordings; we concatenate only the annotations from the 15 sampled 2‐min segments to create the megaclips for evaluation. This strategy yields a total of 50 megaclips (10 per diagnostic group). Unless stated otherwise, all analyses are performed on these megaclips.

## 3 Results

We first compare overall algorithm performance, pooled across all participants, in Section 3.1, examine whether these performance patterns vary by diagnostic group in Section 3.2, before exploring other predictors of performance in Section 3.3.

### 3.1 Performance Comparison Between LENA and ACLEW

Before we turn to a comparison between LENA and ACLEW algorithms, we provide summary statistics from human annotations to help readers understand the types of interactions captured in our 30‐min megaclips (Table 4). On average, megaclips contained 6.6 min of speech/vocalizations (summing across all speaker categories), with the lowest volubility clip containing only 1.3 min of speech and the highest containing 13.6 min of speech, or a third of the megaclip duration. Adult female speech dominated these interactions, accounting for 40.6% of vocalization time (range: 10.9%–75.4%), closely followed by key child vocalizations at 33.0% (range: 4.0%–60.8%). In comparison, other children and adult males contribute at 13.5% (range: 0.0%–39.1%) and 13.0% (range: 0.0%–72.4%), respectively. In terms of measures, each megaclip averaged 93.2 conversational turns between the key child and an adult (range: 0–274), 827.2 adult words (range: 142–1991), and 142.5 speech‐like vocalizations from the key child (range: 7–312).

**TABLE 4:** Summary statistics of speech/vocalization measures found in our fifty 30‐min megaclips according to human annotation.

Speech/Vocalization measures	Mean	Min	Max
Cumulated speech/vocalization duration (min)	6.6	1.3	13.6
Key child's vocalizations (%)	33.0	4.0	60.8
Other children's vocalizations (%)	13.5	0.0	39.1
Adult female speech (%)	40.6	10.9	75.4
Adult male speech (%)	13.0	0.0	72.4
Conversational turn count	93.2	0.0	274.0
Adult word count	827.2	142.0	1991.0
Child vocalization count	142.5	7.0	312.0

*Note*: Speaker category proportions represent each category's relative contribution to the total speech/vocalization time.

With a better understanding of the quantity of interactions captured in our megaclips, we now turn to the performance comparison between LENA and ACLEW.

#### 3.1.1 Segmentation Into Speaker Categories: Identification Error Rate and Percentage Correct

**FIGURE 2:**
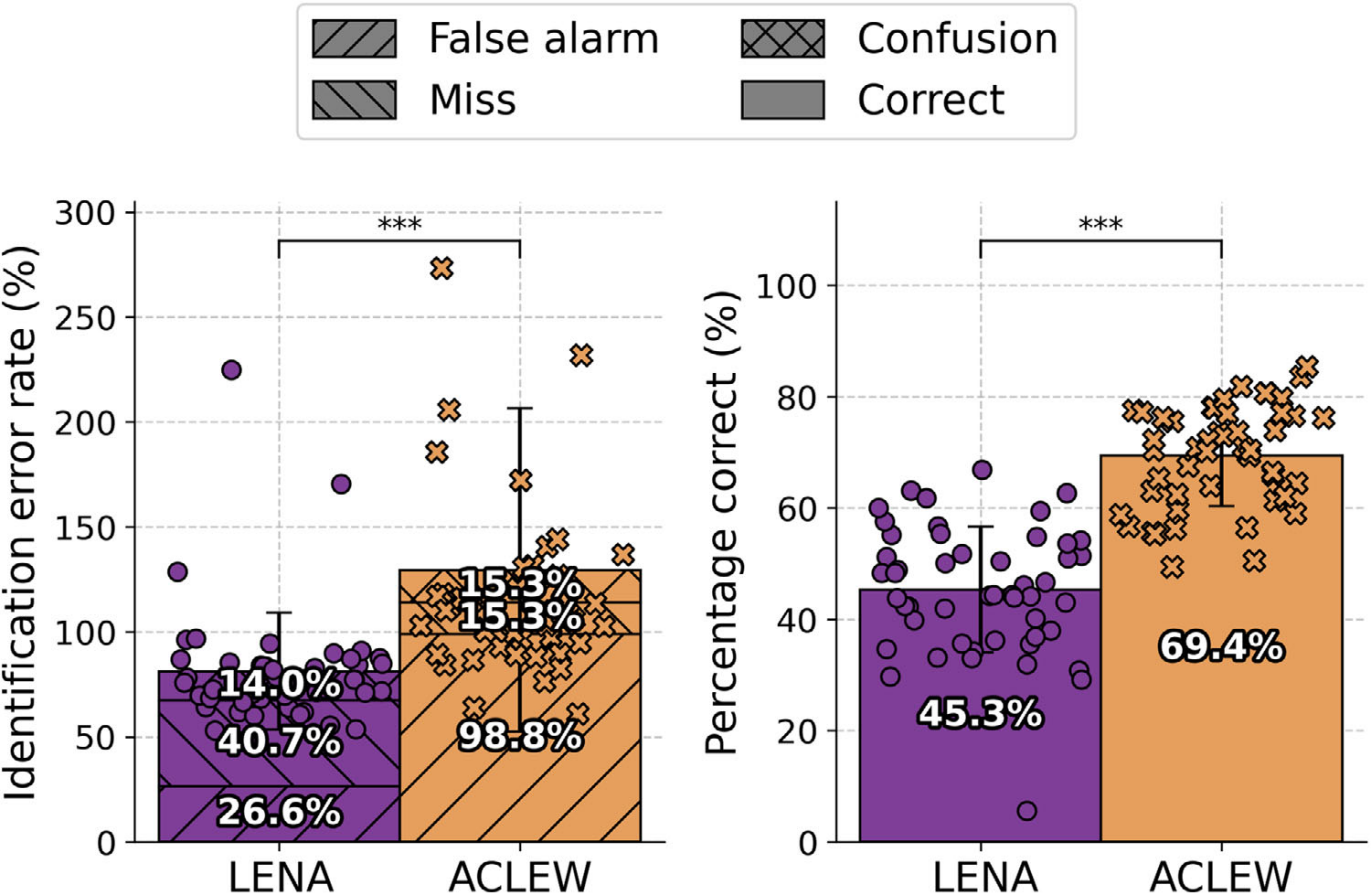
Identification error rate computed as the sum of false alarm, miss, and confusion rates (lower is better) and percentage correct (higher is better) obtained by the LENA and ACLEW algorithms. Each point represents the performance on a 30‐min megaclip (*n* = 50), and error bars represent the mean and standard deviation across megaclips. Two‐tailed Wilcoxon signed‐rank tests were used to determine which algorithm performed significantly better (*p < 0.05, **p < 0.01, ***p < 0.001). Two outliers with high identification error rates (528% from the Fragile X group and 351% from the low‐risk group) for ACLEW are not displayed in the left graph to preserve visibility of the main distribution.

In Figure 2, we compare the overall segmentation performance of the LENA and ACLEW algorithms. All descriptive statistics are reported as mean (*M*), standard deviation (SD), median (*Mdn*), and interquartile range (*IQR*) for key performance metrics. Results show that LENA has a significantly lower identification error rate (*M* = 81.3%, SD = 27.6%, *Mdn* = 76.3%, *IQR* = 15.5%) than ACLEW (*M* = 129.4%, SD = 76.1%, *Mdn* = 109.6%, *IQR* = 28.1%), Wilcoxon signed‐rank test statistic *W* = 1.0*, p* < 0.001. The breakdown of error types reveals distinctly different failure modes between the two systems. LENA's errors are dominated by misses (40.7%), meaning it fails to detect about 41 h of every 100 h of actual speech, while false alarms (26.6%) and confusion errors (14.0%) occur less frequently. In contrast, while ACLEW has relatively low rates of misses (15.3%) and confusion errors (15.3%), its primary weakness is false alarms (98.8%): For every 100 h of actual speech in the recordings, ACLEW incorrectly flags an additional 99 h of nonspeech as speech.

ACLEW achieved significantly higher percentage correct (*M* = 69.4%, SD = 8.9%, *Mdn* = 70.6%, *IQR* = 14.2%) than LENA (*M* = 45.3%, SD = 11.2%, *Mdn* = 44.4%, *IQR* = 16.0%), *W* = 0.0, *p* < 0.001, meaning that out of 100 h of speech/vocalizations found by the human annotator, ACLEW correctly identifies who is speaking for approximately 69 h, while LENA correctly identifies only 45 h.

Overall, this analysis reveals two different segmentation approaches: LENA makes fewer errors but misses more speech, while ACLEW successfully detects more speech but at the cost of more false detections.

#### 3.1.2 Segmentation Into Speaker Categories: Confusion Matrices

**FIGURE 3:**
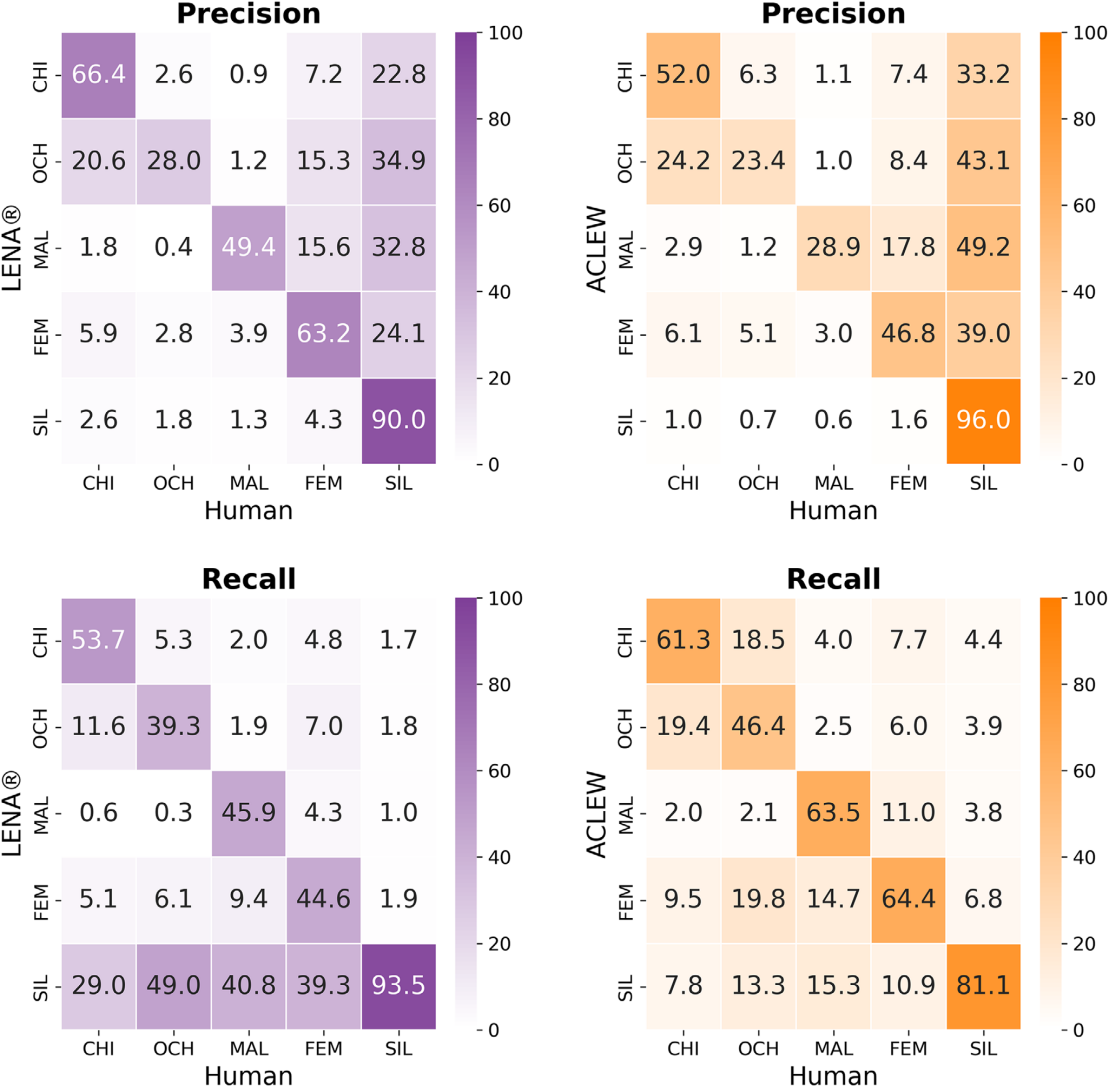
Comparison of LENA and ACLEW algorithms' performance using confusion matrices. The top row of matrices displays precision (accuracy of predictions), with matrices normalized by the total duration each algorithm identified per category. The bottom row of matrices depicts recall (proportion of actual durations detected per category), with matrices normalized by the true duration per category. LENA's results are shown in the left column of matrices, and ACLEW's in the right. For both precision and recall, a higher number indicates better performance. Speaker categories are CHI for key‐child vocalizations, OCH for vocalizations from any other children, MAL for male adult speech, FEM for female adult speech, and SIL for silence/nonspeech.

Having analyzed overall segmentation performance metrics, we now examine how each algorithm confuses the different categories using the confusion matrices reported in Figure 3.

LENA's precision matrix shows that when it detects key child vocalizations, it is correct 66.4% of the time. However, its recall matrix reveals two main error patterns. First, it misclassifies child vocalizations as silence: Out of 100 h of key child vocalizations, 29 h are labeled as silence, and for other children's vocalizations, this rises to 49 h. Second, it shows moderate confusion between child categories, labeling 11.6 h of key child vocalizations as other children and 5.3 h of other children's vocalizations as key child per 100 h. When LENA identifies adult speech, it shows good precision (male: 49.4%, female: 63.2%) but detects only 44–46 h out of every 100 h of adult vocalizations.

ACLEW takes a different approach. When it classifies a segment as the key child, it is correct 52% of the time. Its recall is higher: Out of 100 h of key child vocalizations, it correctly identifies 61.3 h, with some confusion between child categories: 19.4 h of key child vocalizations are labeled as other children, and conversely, 18.5 h of other children's vocalizations are classified as key child. The system also shows notable confusion between other children and female adults: For 100 h of other children's speech, 19.8 h are classified as female speech. For adult speech, while ACLEW detects more vocalizations (64.4 h out of 100), it does so with lower precision (male: 28.9%, female: 46.8%) and shows confusion between male and female categories: For 100 h of male speech, 14.7 h are labeled as female, and for 100 h of female speech, 11.0 h are labeled as male. Both systems perform most reliably on silence (i.e., nonspeech) detection (LENA with a precision of 90.0% and a recall of 93.5%, ACLEW with a precision of 96.0% and a recall of 81.1%).

These confusion matrices provide deeper insight into the different strategies employed by the two algorithms. LENA's conservative approach of classifying uncertain segments as silence leads to higher precision but lower recall. In contrast, ACLEW attempts to classify more segments rather than defaulting to silence, achieving higher recall but inevitably creating more false alarms. Both algorithms show their best performance for key child vocalizations, followed by female adult speech, male adult speech, with other children's vocalizations being the most challenging to detect accurately.

#### 3.1.3 Automatic Counts (CTC, AWC, CVC): Error and Percentage Error

**FIGURE 4:**
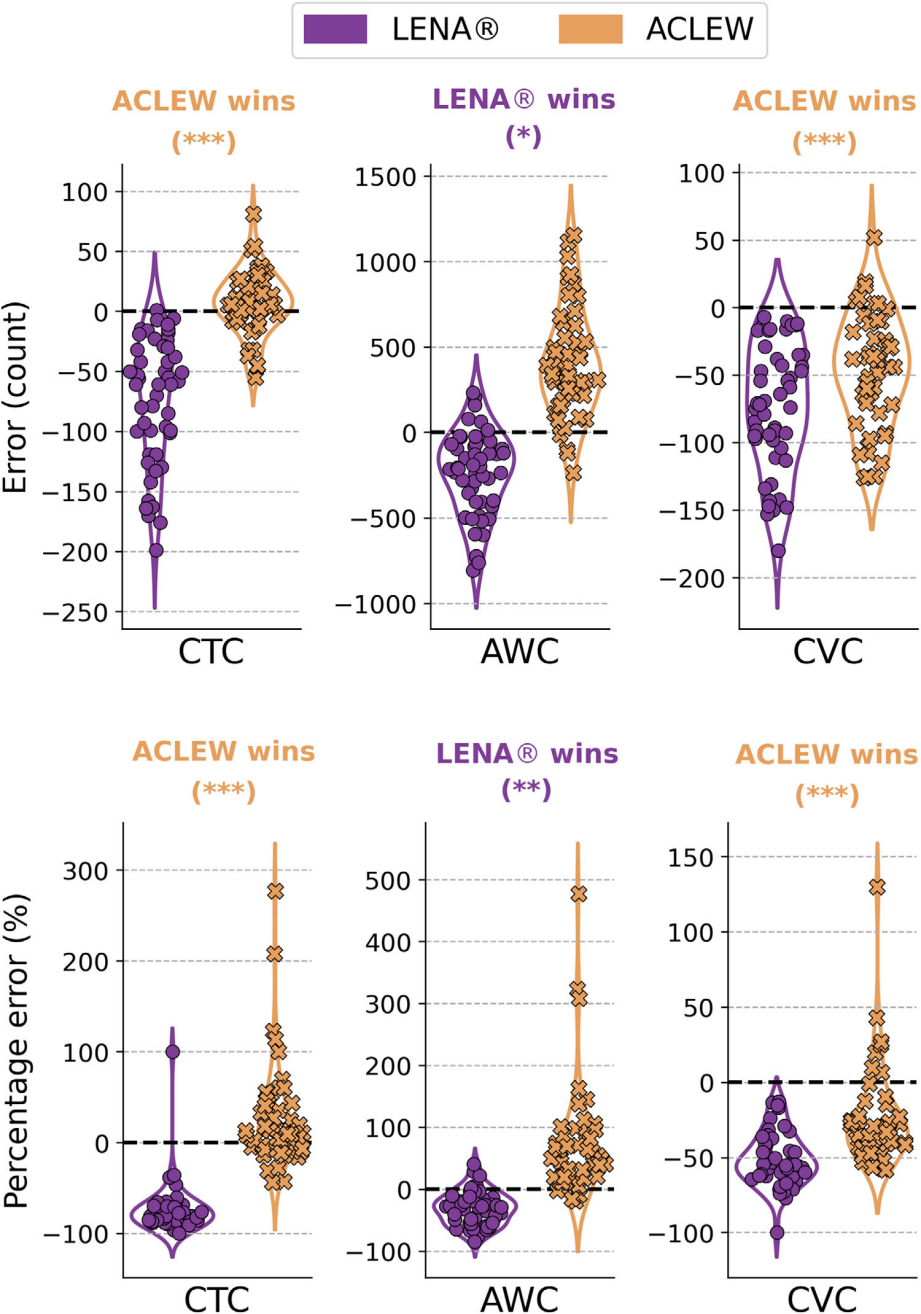
Violin plots showing measurement errors (top) and percentage errors (bottom) between automated (LENA and ACLEW) and human annotations for conversational turn count (CTC), adult word count (AWC), and child vocalization count (CVC). Each point represents a 30‐min megaclip (*n* = 50). Horizontal dashed lines indicate zero error. Two‐tailed Wilcoxon signed‐rank tests on absolute errors were used to determine which algorithm performed significantly better (*p < 0.05, **p < 0.01, ***p < 0.001).

Having examined the segmentation patterns of both algorithms, we now evaluate their performance on three automatic counts commonly used in research: CTC, AWC, and CVC. Before presenting these results, it is important to consider how segmentation errors affect downstream measures. A critical point is that any segment not detected during the initial segmentation step is permanently lost for subsequent analyses. For example, a correctly identified key‐child vocalization followed by a missed female adult segment will not count towards CTC. In contrast, some segmentation errors will not necessarily affect downstream analyses. For instance, a male adult segment misattributed to the female adult category will not impact AWC since both are adults. Similarly, if this misattributed segment is followed by a correctly identified key‐child segment, it will correctly count towards CTC. These observations reveal that a high precision/low recall segmentation strategy may not necessarily be optimal for downstream analyses. With these considerations in mind, we now examine how LENA and ACLEW perform on CTC, AWC, and CVC measures in Figure 4.

Looking first at CTC, LENA underestimates by about 73.0 turns per recording on average, corresponding to predictions 73.7% below true values (in counts: SD = 51.9, *Mdn* =−59.5, *IQR* = 71.5; in %: SD = 28.0%, *Mdn* = −81.0%, *IQR* = 15.6%). ACLEW overestimates CTC by 6.9 turns per megaclip on average, corresponding to predictions 26.7% above true values (in counts: SD = 25.6, *Mdn* = 6.0, *IQR* = 28.3; in %: SD = 56.7%, *Mdn* = 10.1%, *IQR* = 47.5%). ACLEW is significantly more accurate than LENA in estimating CTC (*W* = 90.0, *p* < 0.001 for absolute errors; *W* = 130.5, *p* < 0.001 for absolute percentage errors). The outliers with ACLEW's high percentage error (200%–275%) correspond to megaclips dominated by environmental sounds (same as in Figure 2), where TV speech is misclassified as adult speech.

For AWC, both algorithms show substantial errors but in opposite directions. LENA underestimates by an average of 234.5 words, corresponding to predictions 30.4% below true values (in counts: SD = 238.2, *Mdn* = −207.7, *IQR* = 332.6; in %: SD = 27.1%, *Mdn* = −28.2%, *IQR* = 38.8%), while ACLEW overestimates by 407.7 words, corresponding to predictions 72.0% above true values (in counts: SD = 312.7, *Mdn* = 373.1, *IQR* = 329.7; in %: SD = 87.6%, *Mdn* = 51.7%, *IQR* = 68.9%). This pattern is expected and is a direct consequence of LENA's high precision/low recall strategy (missing some valid segments) and ACLEW's low precision/high recall strategy (including some invalid nonspeech segments). Here, we again see the outliers on which ACLEW obtains a high percentage error. Overall, LENA is significantly more accurate than ACLEW in estimating AWC (*W* = 410.0, *p* = 0.028 for absolute errors; *W* = 339.0, *p* = 0.003 for absolute percentage errors).

Both algorithms underestimate CVC, though to different degrees. LENA misses about 73.3 vocalizations on average, with predictions 51.1% below true values (in counts: SD = 45.9, *Mdn* = −71.5, *IQR* = 62.0; in %: SD = 17.5%, *Mdn* = −54.6%, *IQR* = 20.7%), while ACLEW misses 47.3 vocalizations on average, with predictions 24.6% below true values (in counts: SD = 41.5, *Mdn* = −43.0, *IQR* = 52.5; in %: SD = 31.3%, *Mdn* = −31.7%, *IQR* = 17.7%). ACLEW is significantly more accurate than LENA in estimating CVC (*W* = 164.5, *p* < 0.001 for absolute errors; *W* = 142.0, *p* < 0.001 for absolute percentage errors).

In summary, each algorithm shows distinct strengths. ACLEW excels at estimating conversational turns, providing significantly more accurate CTC values than LENA's consistent underestimates. For AWC, LENA demonstrates better performance despite its tendency to underestimate, while ACLEW's overestimation leads to larger absolute errors. Finally, in CVCs, both algorithms underestimate the true values, though ACLEW achieves significantly better accuracy than LENA.

#### 3.1.4 Automatic Counts (CTC, AWC, CVC): Pearson's *R* Coefficients

**FIGURE 5:**
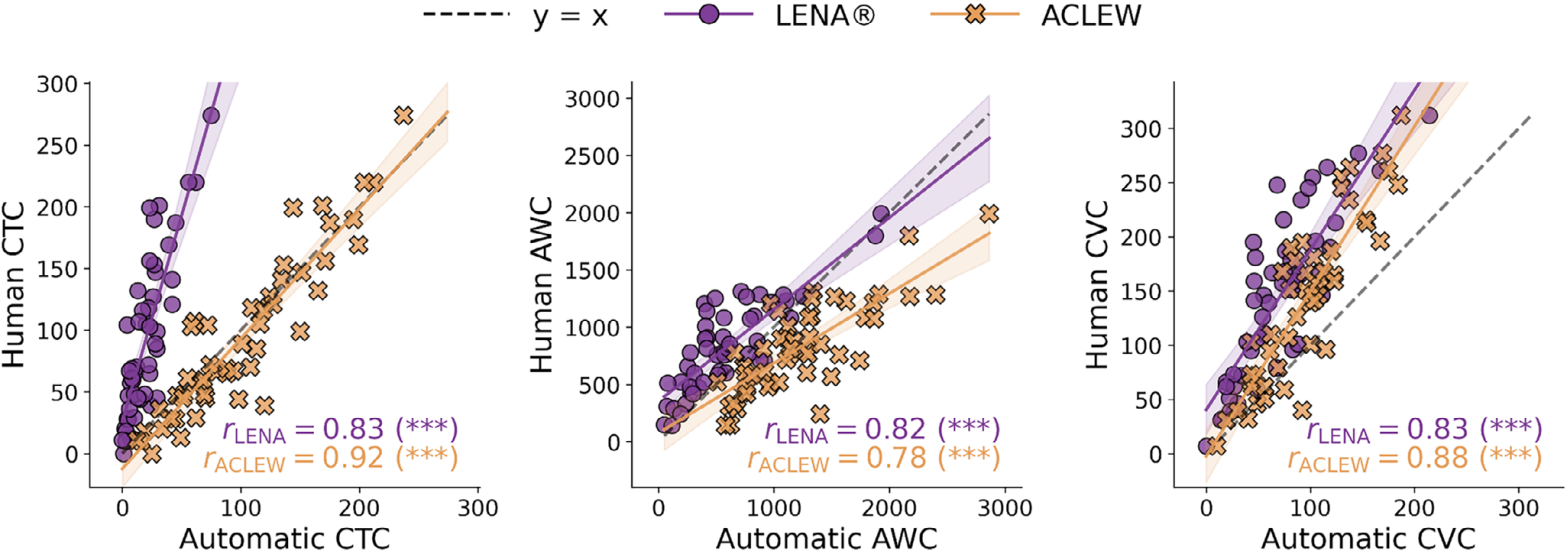
Scatter plots comparing automated and human annotations for conversational turn count (CTC), adult word count (AWC), and child vocalization count (CVC) using LENA (purple circles) and ACLEW (orange crosses) algorithms. The black dashed line (*y = x*) represents perfect agreement between human and automatic counts. Each point represents a 30‐min megaclip (*n* = 50). Solid purple and orange lines show linear regression fits for LENA and ACLEW, respectively, with shaded 95% confidence intervals. Pearson's *r* correlation coefficients with significance levels (***p < 0.001) are shown on the bottom right.

While error metrics assess the accuracy of automatic counts, they do not capture how well the algorithms track relative differences between recordings—a key consideration when comparing across conditions or tracking changes over time. In Figure 5, we report Pearson's correlation coefficients (*r*) to assess the linear relationship between automatic and human counts, regardless of systematic errors in absolute values.

Overall, both algorithms effectively capture a substantial proportion of the variance in human counts, with Pearson's correlation coefficients ranging from 0.78 to 0.92 (corresponding to *R*‐squared values of 60%–84%). For CTC, ACLEW achieves a higher correlation (*r* = 0.92) than LENA (*r* = 0.83), and its measurements align more closely along the *y = x* line (consistent with Figure 4). Both algorithms demonstrate robust correlations for AWC, with LENA achieving *r* = 0.82 and ACLEW *r* = 0.78. For CVC, correlations were *r* = 0.83 for LENA and *r* = 0.88 for ACLEW.

The Pearson's *r* values we found for LENA AWC (*r* = 0.82) and CVC (*r* = 0.83) are compatible with Cristia et al.’s (2020) systematic review, which reported mean correlations of 0.79 for AWC and 0.77 for CVC. However, our LENA CTC correlation (*r* = 0.83) is markedly higher than their reported mean of 0.36, which was based on six studies with highly variable Pearson's *r* (ranging from −0.03 to 0.70). Despite reviewing these papers, we could not identify specific methodological factors explaining such exceptionally low correlations. At present, our study does not support the view that LENA's CTC estimate is unreliable, suggesting that it is important to understand why previous studies found such variability and to identify potential failure cases of LENA.

### 3.2 Investigating Biases Across Diagnostic Groups

We now turn to our second question: “Do LENA and ACLEW perform equally well across diagnostic groups?” This question is crucial, as systematic differences in algorithm performance could lead to unreliable measurements in specific populations and potentially biased research and clinical conclusions about language development in children with neurodevelopmental conditions.

Before we start, we provide group‐level summary statistics in Table 5 to help readers understand how child–caregiver interactions may vary across diagnostic groups. Note that, unlike Section 3.1, the subsequent analyses use linear mixed models applied to 2‐min clips rather than 30‐min megaclips, as the larger number of observations (750 vs. 50) provides greater statistical power to detect potential group differences. These statistics reflect the types of interactions captured in our clips but may not necessarily generalize to other datasets or contexts. We present these descriptive statistics to provide context for the subsequent algorithm performance analyses, rather than to draw conclusions about group differences.

**TABLE 5:** Group‐level summary statistics of speech/vocalization measures found in our 2‐min clips (15 per child, 150 per group) according to human annotation.

Speech/Vocalization measures	Low risk			Angelman syndrome			Down syndrome		
	Mean	Min	Max	Mean	Min	Max	Mean	Min	Max
Cum. speech duration (s)	26.3	0.0	120.2	25.1	0.0	103.8	28.3	0.0	104.3
Key child's vocalizations (%)	48.3	0.0	100.0	34.6	0.0	100.0	32.0	0.0	100.0
Other children's vocalizations (%)	14.4	0.0	62.3	13.2	0.0	87.7	18.7	0.0	87.6
Adult female speech (%)	31.2	0.0	100.0	42.9	0.0	100.0	35.2	0.0	100.0
Adult male speech (%)	6.1	0.0	100.0	9.3	0.0	98.0	14.1	0.0	100.0
Conversational turn count (CTC)	6.3	0.0	49.0	6.1	0.0	67.0	5.7	0.0	40.0
Adult word count (AWC)	48.1	0.0	403.0	57.9	0.0	302.0	65.7	0.0	300.0
Child vocalization count (CVC)	9.5	0.0	52.0	7.7	0.0	58.0	9.6	0.0	56.0

Speech/Vocalization measures	Fragile X syndrome			Sibling with autism		
	Mean	Min	Max	Mean	Min	Max
Cum. speech duration (s)	24.9	0.0	123.3	27.0	0.0	106.3
Key child's vocalizations (%)	41.8	0.0	100.0	47.6	0.0	100.0
Other children's vocalizations (%)	5.1	0.0	100.0	10.0	0.0	90.5
Adult female speech (%)	38.5	0.0	100.0	26.9	0.0	98.3
Adult male speech (%)	14.6	0.0	100.0	15.5	0.0	97.2
Conversational turn count (CTC)	6.6	0.0	50.0	6.4	0.0	41.0
Adult word count (AWC)	51.7	0.0	386.0	52.4	0.0	316.0
Child vocalization count (CVC)	9.8	0.0	44.0	11.0	0.0	62.0

*Note*: Speaker category proportions represent each category's relative contribution to the total speech/vocalization time. Note that the cumulated speech duration can go above 120 s due to overlapping speech.

Within our samples, the average cumulative speech duration was fairly consistent across groups, ranging from 24.9 s (Fragile X syndrome) to 28.3 s (Down syndrome) per 2‐min clip. The proportion of key‐child vocalizations ranged from 32.0% (Down syndrome) to 48.3% (Low risk), and that of other children from 5.1% (Fragile X syndrome) to 18.7% (Down syndrome). Adult female speech ranged from 26.9% (siblings with autism) to 42.9% (Angelman syndrome), and adult male speech from 6.1% (Low risk) to 15.5% (siblings with autism). Conversational turns (CTC) ranged from 5.7 (Down syndrome) to 6.6 (Fragile X syndrome), AWCs from 48.1 (low risk) to 65.7 (Down syndrome), and CVCs from 7.7 (Angelman syndrome) to 11.0 (siblings with autism).

#### 3.2.1 Is the Segmentation Into Speaker Categories Equally Reliable Across Diagnostic Groups?

To evaluate whether the segmentation step is equally reliable across diagnostic groups, we ran statistical tests on the performance metrics obtained on our 2‐min clips (150 per diagnostic group; 750 total). We used linear mixed models to account for the nested structure of our data (clips within participants). We conducted two separate models, one predicting miss rate and another predicting percentage correct. Each model included fixed effects of diagnostic group, sex, and age of the key child, with a random intercept for the child. We excluded clips in which human annotators found no speech (keeping *n* = 581 clips, 77.4%), as performance metrics were undefined for these (division by 0).

As reported in Table 6, results showed significant positive intercepts for both miss rates (LENA: *β* = 42.51, SE = 7.80, *z* = 5.45, *p* < 0.001; ACLEW: *β* = 16.43, SE = 5.75, *z* = 2.86, *p* = 0.004) and percentage correct (LENA: *β* = 48.62, SE = 8.48, *z* = 5.74, *p* < 0.001; ACLEW: *β* = 68.89, SE = 7.57, *z* = 9.10, *p* < 0.001). However, we found no significant effects of diagnostic group, sex, or age on either performance metric for both LENA and ACLEW.

**TABLE 6:** Results from two linear mixed models predicting miss rates and percentage correct of LENA and ACLEW segmentation algorithms based on *n* = 581 clips.

Dependent variable		LENA				ACLEW			
	Fixed effect	*β*	SE	*z*	*p*	*β*	SE	*z*	*p*
Miss (%)	Intercept	**42.51**	**7.80**	**5.45**	**<0.001 (** ^***^)	**16.43**	**5.75**	**2.86**	**0.004 (** ^**^)
	AS	4.27	4.69	0.91	0.362	4.11	3.45	1.19	0.233
	FX	−1.10	4.66	−0.24	0.813	3.18	3.43	0.93	0.353
	DS	6.37	4.57	1.39	0.163	5.11	3.37	1.51	0.130
	Asib	−4.33	4.67	−0.93	0.353	0.17	3.44	0.05	0.960
	Sex = male	−0.75	3.02	−0.25	0.805	−1.68	2.23	−0.75	0.452
	Age	−0.08	0.34	−0.23	0.820	−0.07	0.25	−0.30	0.767
Percentage correct (%)	Intercept	**48.62**	**8.48**	**5.74**	**<0.001 (** ^***^)	**68.89**	**7.57**	**9.10**	**<0.001 (** ^***^)
	AS	−4.46	5.08	−0.88	0.381	−2.29	4.54	−0.50	0.615
	FX	−1.29	5.05	−0.26	0.799	−1.24	4.51	−0.28	0.784
	DS	−4.71	4.98	−0.95	0.344	−6.68	4.44	−1.50	0.133
	Asib	−0.02	5.07	0.00	0.997	−0.70	4.53	−0.15	0.876
	Sex = male	0.84	3.29	0.25	0.799	0.37	2.94	0.13	0.900
	Age	−0.12	0.37	−0.32	0.749	0.13	0.33	0.38	0.706

*Note*: Each model included fixed effects for the *diagnostic group* (reference: low risk; groups: Angelman syndrome [AS], Fragile X syndrome [FX], Down syndrome [DS], sibling with autism [Asib]), *sex* (reference: female), and *age*, plus a random intercept for *child id*. For each fixed effect, we report the beta coefficient (*β*), the standard error (*SE*), the *z*‐statistic (*z*), and the *p* value (*p*).

Significant results are marked in bold with asterisks indicating significance levels (*p < 0.05, **p < 0.01, ***p < 0.001).

To evaluate the overall contribution of the diagnostic group, we conducted likelihood ratio tests comparing models with and without the diagnostic group as a predictor (both models included sex and age as fixed effects, plus a random intercept for child_id). These tests confirmed that diagnostic group did not improve model fit for either miss rates or percentage correct in both LENA (miss rates: *χ* ^2^(4) = 4.67, *p* = 0.322; percentage correct: *χ^2^* (4) = 2.85, *p* = 0.584) and ACLEW (miss rates: *χ^2^* (4) = 3.94, *p* = 0.414; percentage correct: *χ^2^* (4) = 2.36, *p* = 0.670). These analyses suggest that LENA and ACLEW's segmentation performance does not systematically vary across diagnostic groups. Similar analyses with the false alarm and confusion rates led to the same conclusion (not reported here for the sake of brevity but available upon request).

#### 3.2.2 Are Automatic Counts (CTC, AWC, CVC) Equally Accurate Across Diagnostic Groups?

We next analyze the level of performance of automatic counts across diagnostic groups. Similar to the previous section, we ran statistical tests on our seven hundred and fifty 2‐min clips to probe for group differences, nesting each clip within child. We ran three linear models, each predicting either CTC_human_, AWC_human_, or CVC_human_. Each model includes fixed effects for the automatic count (CTC_automatic_, AWC_automatic_, or CVC_automatic_), diagnostic group, sex, and age of the key child, with a random intercept of the child. Contrary to the analysis presented in Section 3.2.1, all 2‐min clips were included (150 per group) as all variables remain mathematically defined even when no speech is present (counts are simply equal to 0 in this case). Note, however, that results were similar when excluding clips with no speech. Results are reported in Table 7.

**TABLE 7:** Results from three linear mixed models predicting CTC_human_, AWC_human_, or CVC_human_ based on *n* = 750 clips.

Dependent variable		LENA				ACLEW			
	Fixed effect	*β*	SE	*z*	*p*	*β*	SE	*z*	*p*
CTC_human_	Intercept	0.36	0.50	0.73	0.467	**2.56**	**1.13**	**2.27**	**0.023 (** ^*^)
	CTC_automatic_	**0.16**	**0.01**	**25.12**	**<0.001 (** ^***^)	**0.69**	**0.02**	**39.60**	**<0.001 (** ^***^)
	AS	0.42	0.29	1.42	0.155	−0.32	0.67	−0.48	0.631
	FX	0.46	0.29	1.56	0.118	0.35	0.66	0.53	0.597
	DS	0.46	0.29	1.58	0.115	**1.40**	**0.66**	**2.12**	0.034 (*)
	Asib	0.27	0.30	0.90	0.367	0.11	0.67	0.16	0.871
	Sex = male	−0.04	0.19	−0.23	0.815	0.77	0.44	1.75	0.079
	Age	−0.01	0.02	−0.67	0.500	−0.04	0.05	−0.85	0.395
AWC_human_	Intercept	−8.21	12.23	−0.67	0.502	−4.01	15.60	−0.26	0.797
	AWC_automatic_	**0.61**	**0.02**	**30.79**	**0.001 (** ^***^)	**0.95**	**0.03**	**31.50**	**<0.001 (** ^***^)
	AS	**16.63**	**7.21**	**2.31**	**0.021 (** ^*^)	**19.29**	**9.19**	**2.10**	0.036 (*)
	FX	**19.54**	**7.14**	**2.74**	**0.006 (** ^**^)	5.36	9.10	0.59	0.555
	DS	10.86	7.15	1.52	0.129	9.10	9.12	1.00	0.318
	Asib	**17.97**	**7.24**	**2.48**	**0.013 (** ^*^)	**22.36**	**9.23**	**2.42**	**0.015 (** ^*^)
	Sex = male	3.58	4.74	0.75	0.451	4.96	6.04	0.82	0.411
	Age	−0.04	0.54	−0.08	0.939	0.95	0.69	1.37	0.171
CVC_human_	Intercept	−0.67	1.23	−0.55	0.583	**2.84**	**1.10**	**2.58**	**0.010 (** ^*^)
	CVC_automatic_	**0.43**	**0.01**	**29.07**	**<0.001 (** ^***^)	**0.42**	**0.01**	**29.18**	**<0.001 (** ^***^)
	AS	0.91	0.72	1.25	0.210	−0.30	0.65	−0.46	0.643
	FX	0.06	0.72	0.09	0.931	0.14	0.64	0.22	0.829
	DS	−0.39	0.72	−0.54	0.588	0.55	0.64	0.85	0.393
	Asib	0.81	0.73	1.12	0.263	−0.24	0.65	−0.36	0.718
	Sex = male	0.33	0.48	0.69	0.491	0.76	0.43	1.77	0.077
	Age	0.03	0.06	0.61	0.543	−0.04	0.05	−0.83	0.404

*Note*: Each model included fixed effects for the automatic count (CTC_automatic_, AWC_automatic_, or CVC_automatic_), the *diagnostic group* (reference: low risk; groups: Angelman syndrome [AS], Fragile X syndrome [FX], Down syndrome [DS], sibling with autism [Asib]), *sex* (reference: female), and *age*, plus a random intercept for *child id*. For each fixed effect, we report the beta coefficient (*β*), the standard error (*SE*), the *z*‐statistic (*z*), and the *p* value (*p*).

Significant results are marked in bold with asterisks indicating significance levels (*p < 0.05, **p < 0.01, ***p < 0.001).

Our linear mixed models reveal several important patterns in the relationship between automatic and human counts. First, both LENA and ACLEW's automatic counts are strongly predictive of human counts, as evidenced by highly significant effects (all *p* < 0.001) across all three counts (CTC, AWC, and CVC).

The relationship between automatic and human counts also showed some systematic differences across diagnostic groups. For AWC, after controlling for the automatic count, the human count was systematically higher in children with Angelman syndrome compared to the low‐risk group, a pattern observed with both LENA (*β* = 16.63, SE = 7.21, *z* = 2.31, *p* = 0.021) and ACLEW (*β* = 19.29, SE = 9.19, *z* = 2.10, *p* = 0.036). In other words, when the algorithms produce the same AWC estimate for a child with Angelman syndrome and a low‐risk child, human annotators count approximately 17–19 more adult words for the child with Angelman syndrome. A similar pattern was observed for siblings of children with autism (LENA: *β* = 17.97, SE = 7.24, *z* = 2.48, *p* = 0.013; ACLEW: *β* = 22.36, SE = 9.23, *z* = 2.42, *p* = 0.015). Only LENA showed this systematic difference for the Fragile X syndrome group (*β* = 19.54, SE = 7.14, *z* = 2.74, *p* = 0.006). For CTC, ACLEW showed a similar pattern in children with Down syndrome, where human counts exceeded automatic predictions by approximately 1.4 conversational turns compared to the low‐risk group (*β* = 1.40, SE = 0.66, *z* = 2.12, *p* = 0.034). The relationship between automatic and human CVC remained consistent across diagnostic groups, with no significant systematic differences detected in either algorithm. Age and sex of the key child had minimal impact on the relationship between automatic and human counts, with no significant effect found.

To better understand these group differences, we ran likelihood ratio tests comparing models with and without diagnostic group as a predictor (both models included the automatic count, sex, and age as fixed effects, plus a random intercept for child_id). These tests revealed that the diagnostic group provided minimal additional predictive value beyond the automatic counts. For LENA, adding a diagnostic group significantly improved model fit only for AWC (*χ* ^2^(4) = 10.42, *df* = 4, *p* = 0.034), but explained just 0.9% of additional variance. For ACLEW, the diagnostic group did not significantly improve model fit for any measure (all *p* > 0.05, though AWC showed a marginal trend with *p* = 0.073), with additional variance explained ranging from 0.2% to 0.6%. These minimal increments in explained variance contrast sharply with the strong predictive power of the automatic counts, which alone explained 51%–70% of the variance in human counts. Thus, while individual coefficient tests identified some statistically significant group differences for specific measures, the overall contribution of the diagnostic group to model prediction is negligible. This suggests that both LENA and ACLEW provide robust estimates across diagnostic groups.

### 3.3 Factors Driving Algorithm Performance Variation

Our investigation revealed remarkably stable performance for both LENA and ACLEW across diagnostic groups. However, while variability between diagnostic groups was negligible, we observed substantial within‐group variability in algorithmic performance. For example, within the low‐risk group alone, LENA's identification error rate ranged from 60% to over 100%, and ACLEW's from 60% to over 200%. This substantial within‐group variability suggests that factors other than diagnostic group membership primarily drive algorithm performance.

To identify these factors, we conducted ordinary least‐squares regressions with performance metrics as dependent variables and human‐annotated speech characteristics as predictors. These predictors included the cumulated duration of speech by different speaker types (FEM, MAL, OCH, CHI), the cumulated duration of overlapping speech, canonical, noncanonical, and cry vocalizations, and manual CTC, AWC, and CVC.

Results, presented in Table 8, reveal complex relationships between human‐annotated speech characteristics and algorithm performance, with effects varying by algorithm and metric. The full models explained between 13.4% and 54.4% of performance variance, indicating that speech characteristics account for a substantial portion of algorithm performance.

**TABLE 8:** Significant predictors of performance metrics for LENA and ACLEW.

Performance metric	Algorithm	Significant predictor	*β*	*sr* ^2^ (%)	*p*	Better performance?	Full model *R* ^2^ (%)
False alarm	LENA	None	—	—	—	—	14.6
(%, ↓)	ACLEW	*FEM_dur_*	−0.35	11.6	0.012 (*)	Yes	33.3
Miss	LENA	None	—	—	—	—	37.3
(%, ↓)	ACLEW	None	—	—	—	—	34.4
Confusion	LENA	*OCH_dur_*	−0.04	17.1	0.004 (^**^)	Yes	27.3
(%, ↓)	ACLEW	None	—	—	—	—	13.4
Percentage	LENA	*FEM_dur_*	0.05	8.5	0.038 (^*^)	Yes	26.5
correct (%, ↑)	ACLEW	None	—	—	—	—	29.4
CTC absolute	LENA	None	—	—	—	—	16.6
percentage error (%, ↓)	ACLEW	*OCH_dur_*	0.29	7.8	0.044 (^*^)	No	27.3
AWC absolute percentage error (%, ↓)	LENA	*OCH_dur_*	0.20	21.4	<0.001 (^***^)	No	54.4
		*FEM_dur_*	−0.10	10.1	0.005 (^**^)	Yes	
		*MAL_dur_*	−0.14	8.4	0.010 (^**^)	Yes	
	ACLEW	*OCH_dur_*	−0.67	14.9	0.001 (^**^)	Yes	48.9
		*FEM_dur_*	−0.42	12.4	0.003 (^**^)	Yes	
CVC absolute percentage error (%, ↓)	LENA	None	—	—	—	—	14.6
	ACLEW	*FEM_dur_*	−0.09	10.8	0.024 (^*^)	Yes	22.0
		*OCH_dur_*	−0.12	9.8	0.031 (^*^)	Yes	

*Note*: An ordinary least‐squares regression is used to fit *y* (the performance metric) given *x* (speech/vocalizations measures derived from human annotation). *x* includes*FEM_dur_*, *MAL_dur_*, *OCH_dur_*, *CHI_dur_* (the cumulated duration of segments produced by female adults, male adults, other children, or the key child), $OVL_{dur}$ (the cumulated duration of overlapping speech), $CHI_{dur}^{can}$, $CHI_{dur}^{nonc}$, $CHI_{dur}^{cry}$ (the cumulated duration of canonical, noncanonical, or cry vocalizations produced by the key child), *CTC*, *AWC*, and *CVC*. Type II ANOVA was used to assess the unique contribution of each predictor while controlling for all others in the model. Only predictors explaining a statistically significant (*p* < 0.05) portion of variance are reported. For each significant predictor, we report the regression coefficient (*β*), semi‐partial correlation squared (*sr^2^*), which indicates the percentage of total variance uniquely explained by that predictor, the *p* value, and the significance level with *p ≤ 0.05, **p ≤ 0.01, ***p ≤ 0.001. The “Better performance?” column indicates whether an increase in that predictor leads to better algorithm performance (i.e., moves the performance metric in the desired direction).

For LENA, female adult speech duration (*FEM_dur_*) consistently predicted better performance, being associated with a higher percentage correct (*β* = 0.05, *sr* ^2^ = 8.5%, *p* = 0.038) and lower AWC absolute percentage error (*β* = −0.10, *sr* ^2^ = 10.1%, *p* = 0.005). Male adult speech duration (*MAL_dur_*) also reduced AWC errors (*β* = −0.14, *sr* ^2^ = 8.4%, *p* = 0.010). Conversely, other children's speech duration (*OCH_dur_*) showed mixed effects: While it reduced confusion rates (*β* = −0.04, *sr* ^2^ = 17.1%, *p* = 0.004), it increased AWC estimation errors (*β* = 0.20, *sr* ^2^ = 21.4%, *p* < 0.001). For ACLEW, female adult speech duration again proved beneficial, reducing both false alarms (*β* = −0.35, *sr* ^2^ = 11.6%, *p* = 0.012) and AWC estimation errors (*β* = −0.42, *sr* ^2^ = 12.4%, *p* = 0.003). Other children's speech duration showed mixed effects: While it increased CTC estimation errors (*β* = 0.29, *sr* ^2^ = 7.8%, *p* = 0.044), it reduced both AWC (*β* = −0.67, *sr* ^2^ = 14.9%, *p* = 0.001) and CVC estimation errors (*β* = −0.12, *sr* ^2^ = 9.8%, *p* = 0.031). Notably, none of the key‐child predictors emerged as significant predictors of algorithm performance across any of the metrics tested. However, individual effects should be interpreted with caution, given potential correlations among predictors.

These findings suggest that the total speaking time of surrounding speakers (other children and adults), rather than the diagnosis of the key child, primarily drives algorithmic performance variation.

## 4 Discussion

**Findings summary**. Our comparison of the LENA and ACLEW algorithms across diverse neurodevelopmental profiles revealed differences in algorithm performance that were relatively consistent across diagnostic groups. Regarding the audio segmentation into speaker categories, LENA makes relatively few mistakes but misses many segments, whereas ACLEW retrieved more speech/vocalizations but at the cost of more false alarms. To illustrate this contrast with concrete numbers: for every 100 h of speech/vocalizations found by our human expert, LENA correctly classified 45 h (assigning the correct speaker category), missed 41 h entirely (outputting nonspeech when there was speech), generated 27 h of false alarms (detecting speech when there was none), and confused the speaker category for 14 h. ACLEW, by comparison, correctly classified 69 h out of those same 100 h, missing only about 15 h, confused the speaker category for approximately 15 h, but generated 99 h of false alarms. We found lower performance for male adults and other children segments compared to key child and female adult segments (see Section 3.1.2), as documented by Cristia et al. (2021) for LENA and Lavechin et al. (2020) for ACLEW.

For CTC between the key child and an adult, ACLEW demonstrated substantially better accuracy with predictions about 26% above true counts (overestimating by about 7 turns per megaclip), compared to LENA's underestimation by 74% (missing about 73 turns). For AWC, both algorithms showed substantial absolute errors but in opposite directions: LENA underestimated by an average of 234 words (30% below true counts), while ACLEW overestimated by 407 words (72% above true values). Both algorithms underestimated CVC, with ACLEW's estimate 25% below true counts (missing about 47 vocalizations per megaclip) compared to LENA's 51% underestimation (missing about 73 vocalizations). Finally, correlation analyses revealed that both algorithms achieved relatively high Pearson's coefficients when compared with human coding across all counts, with ACLEW showing slightly higher values for CTC (*r* = 0.92 vs. LENA's 0.83) and CVC (*r* = 0.88 vs. 0.83) and LENA showing slightly higher correlation with human counts for AWC (*r =* 0.82 vs. ACLEW's 0.78). These correlation scores suggest that despite errors, both systems effectively capture relative differences in speech patterns within our corpus. However, these strong correlations should be interpreted with caution, as they reflect the strength of the linear association between automatic and human counts, which is directly influenced by the degree of interchild variability. Our corpus likely contains more variability than most corpora studied in the literature because we included diverse neurodevelopmental profiles. Consequently, LENA and ACLEW might show lower correlation in more homogeneous samples consisting only of typically developing children, where subtle differences between children become harder to detect.

Regarding algorithmic biases, we found little evidence of systematic differences in performance for both LENA and ACLEW algorithms across diagnostic groups. Linear mixed models showed no significant effects of diagnostic group on segmentation performance metrics, with only minor systematic differences in the relationship between automatic and human counts. For instance, both algorithms showed slightly lower predictive power on AWC for children with Angelman syndrome and Fragile X syndrome compared to the low‐risk group. Although it is challenging to provide precise evidence for why, we hypothesize that the lower predictive power stems from the higher proportion of male speech in recordings from the Angelman syndrome and Fragile X syndrome cohorts (see Table 5), as both algorithms struggle with detecting male speech. Regarding CVC measurements, despite our expectations of potential biases, the relationship between automatic and human CVC remained consistent across diagnostic groups, with no significant systematic differences detected in either algorithm. While the diagnostic group of the key child did not predict algorithm performance, the total speaking time of surrounding speakers (other children and adults) was a strong predictor of performance.

As algorithms become more robust to challenging recording conditions, it will be important to monitor whether performance improvements occur uniformly across populations. If performance disparities were to emerge, targeted fine‐tuning of open‐source algorithms like ACLEW or others on annotated data from clinically diverse populations could address these issues and ensure stable performance across populations. Additionally, it is important to note that while differences between the two algorithms are small, these differences, however small, may have a greater impact on recordings from children with neurogenetic syndromes if they have very low volubility (e.g., since LENA misses more, it is more likely to struggle to capture change in children who utter very few utterances). Further, differences we observed between algorithms may change over development if, for example, children with neurogenetic syndromes show more distinct patterns from low‐risk children at an older age than we tested.

**Which to choose between LENA and ACLEW?** Making an informed choice will likely require looking at *implementation constraints* (hardware, programming expertise, access to computational resources) and *performance considerations* (algorithm accuracy).

Regarding implementation constraints, LENA allows researchers and practitioners to both collect and analyze data through an easy‐to‐use interface requiring no programming skills. However, the LENA solution is quite costly (count several thousand dollars for initial setup and yearly subscription fees, LENA SP Pricing Document 2025). In contrast, the ACLEW algorithm is entirely free and usable with any recordings, enabling researchers to use the equipment of their choice (e.g., USB recorders as in Scaff et al. 2024 or GoPro head‐mounted cameras as in Long et al. 2023). However, ACLEW requires some programming skills to install dependencies and run the models, typically involving command‐line operations and familiarity with Python environments. This accessibility barrier may limit ACLEW's adoption among clinicians and researchers without programming expertise, though future development of user‐friendly interfaces could help democratize access to open‐source tools. Perhaps another consideration is the running time: ACLEW's segmentation step, the most computation‐intensive phase of the pipeline, takes approximately 1/4th of the audio time on Intel Xeon Gold 6230 CPUs (accessible on typical consumer‐grade laptops) and 1/35th real‐time on an NVIDIA Tesla V100 GPU. If data are processed on a computing cluster, these processing times can be significantly reduced through parallel processing of multiple audio files simultaneously. Whenever possible, we recommend that researchers inquire about computational resources available at their institution, as some universities offer access to high‐performance computing that can accelerate processing. Although the learning curve may seem steep, the skills gained by integrating ACLEW and similar machine learning tools into one's research toolkit will likely transfer to other research projects, especially since these tools are likely to persist and continue to transform developmental and speech sciences.

Regarding performance considerations, the largest difference we observed between LENA and ACLEW lies in their segmentation strategy. Since LENA's false alarm and confusion rates are low (it rarely detects vocalizations that are not actually present and rarely misclassifies one speaker category for another), it is especially suited for creating speech/vocalization datasets with minimal noise or misclassifications (e.g., for further acoustic analyses like pitch). However, LENA's low percentage correct (only around 45% of the true speech duration is correctly retrieved) means such a dataset may not represent the full range of language use. For instance, LENA might miss less distinct vocalizations (less clearly articulated, lower in volume, or with atypical prosodic patterns), which can be problematic when studying populations with atypical language development who may produce less clear speech due to lags in motor skill and/or differences in facial morphology. Further acoustic and linguistic analysis of these missed vocalizations is needed to determine which specific features cause them to go undetected by LENA. While our analysis (Section 3.2.1) suggests that LENA's miss rate is consistent across our five diagnostic groups, the content of what's being missed could still contain diagnostically relevant differences. On the contrary, ACLEW's high percentage correct (it rarely fails to detect vocalizations that are actually present) makes it suited for creating datasets that better capture the full variability of language use. This comes at the price of a high false alarm rate, which introduces a considerable number of misclassifications when environmental sounds are classified as speech. For acoustic analyses, users may want to run an additional pass over segments detected by ACLEW to filter out these nonspeech segments. Given the current level of performance of both algorithms in detecting male speech and other children's vocalizations, we recommend caution when using either system for research questions involving these speaker categories, as already suggested by Cristia et al. (2021)—but see Kunze et al. (2025) and Charlot et al. (2025) for recent improvements on detecting male adult and other children's segments.

In most cases, it is extremely thorny to predict how the algorithm's behavior will impact downstream analyses. For this reason, and under the assumption that implementation constraints can be solved, we recommend using both LENA and ACLEW (when possible) for analyzing daylong recordings, as recommended in Gautheron et al. (2025). Findings can then be interpreted in light of the performance considerations our study has identified for each algorithm. This approach helps ensure robust scientific conclusions by comparing effect sizes and observations across both algorithms.

**Recommendations on validating algorithms**. Finally, we conclude with a set of recommendations for future algorithm validation studies, building on the present study.

Since we annotated 2‐min clips, it may seem logical to compute performance metrics on these same 2‐min clips. Examining the extensive literature on LENA validation studies reveals that researchers typically employ varying clip lengths. For instance, Cristia et al. (2021) compute performance metrics on 2‐min clips; Busch et al. (2018), McDonald et al. (2021), and Bruyneel et al. (2021) used 5‐min clips; Canault et al. (2016) and Ganek and Eriks‐Brophy (2018a, b) used 10‐min clips; and Oetting et al. 2009 used 30‐min clips.

Within a fixed annotation budget, computing performance metrics on a few long clips may be problematic because we risk over‐ or undersampling child–caregiver interactions that are easier or harder for the algorithm to process, for example, storytelling or outdoor sessions. While numerous short clips may better represent the child's entire day, performance metrics on short clips are also problematic because they prevent observing error compensation, whereby local under‐ and overestimations cancel out. We argue that neither solution is fully satisfactory, as performance metrics derived from either approach may not generalize well to the entire daylong recording, which is ultimately what researchers are interested in.

In Figure S1, we compare Pearson's r for LENA and ACLEW's CTC estimate over different timescales, for example, our seven hundred and fifty 2‐min clips as in Cristia et al. (2021) versus fifty 30‐min megaclips as in the present study. We found that correlations between human and automatic counts improve substantially with longer timescales. For instance, LENA's CTC estimate shows a Pearson's *r* of 0.70 on 2‐min clips versus 0.83 on 30‐min megaclips, despite analyzing the exact same underlying audio data.

Whenever possible, we encourage other researchers to follow a similar approach to the one used in this paper by computing performance metrics on megaclips, aggregating multiple short annotated clips per child. This method preserves the diversity of short clips while allowing for error compensation, leading to more reliable and generalizable estimates of performance.

**Limitations**. This study has three primary limitations. First, our use of a single annotator may introduce some subjectivity in annotation decisions, though this is mitigated by the annotator's extensive experience and use of a validated protocol. Importantly, the maximum reported interannotator agreement for this type of data (Cohen's *κ* = 0.64; Cristia et al. 2021) represents a practical ceiling for algorithm performance, as algorithms trained to reproduce human annotations cannot be expected to exceed the level of agreement between human annotators themselves. Both LENA and ACLEW's observed performance should therefore be interpreted relative to this ceiling of human agreement rather than perfect ground truth. Second, the sample size of 10 children per diagnostic group limits statistical power to detect subtle performance differences across neurodevelopmental profiles. While our statistical analyses on seven hundred and fifty 2‐min clips provided additional granularity, our findings can only be reliably generalized to the 50 children in our sample. Larger cohorts, replication studies, and meta‐analyses will be necessary for a more comprehensive validation of both the LENA and ACLEW algorithms. Third, our analysis was restricted to 2‐year‐olds growing up in English‐speaking households based in the United States. Algorithm performance may vary across different ages, languages, and cultural settings, particularly when applied to children with diverse neurodevelopmental profiles. Future validation studies should address this diversity to ensure these tools serve all children who might benefit from them.

## 5 Conclusions

Our comparison of the LENA and ACLEW algorithms across diverse neurodevelopmental profiles yields several insights for researchers and clinicians studying early language environments. While each system demonstrated distinct strengths, ACLEW excelling at conversational turn detection and achieving higher correlations with human measurements on CTC and CVC, LENA showing more conservative but precise speaker classification and providing more accurate estimates of AWC, perhaps the most significant finding was the consistent performance across diagnostic groups.

Despite these algorithms being primarily trained on typically developing children, our analysis revealed the same level of performance when applied to recordings from children with Down syndrome, Fragile X syndrome, Angelman syndrome, or siblings of children with autism. The absence of significant performance differences across diagnostic groups suggests that both LENA and ACLEW can be reliably deployed for studying language environments in 2‐year‐old children with the neurodevelopmental profiles studied here. However, we caution that our sample size per diagnostic group was modest (only 10), and so was our number of clinical groups (only 4). Further validation on larger and more diverse samples would strengthen our findings.

We strongly advocate for a “biased until proven otherwise” position when applying these algorithms to new populations: Machine learning systems inevitably reflect the data on which they were trained and should not be presumed to perform equally well across all populations without empirical validation. We recommend researchers applying these technologies across neurodevelopmental profiles, languages, sociocultural settings, or socioeconomic status to conduct similar validation studies, especially when these populations may be underrepresented in algorithm development and testing.

## Author Contributions

**Marvin Lavechin**: conceptualization, investigation, writing – original draft, methodology, validation, visualization, writing – review and editing, software, data curation, formal analysis, resources. **Lisa R. Hamrick**: conceptualization, investigation, writing – review and editing, funding acquisition, methodology, supervision, project administration, resources. **Bridgette Kelleher**: supervision, conceptualization, investigation, funding acquisition, methodology, writing – review and editing, project administration, resources. **Amanda Seidl**: supervision, conceptualization, investigation, funding acquisition, methodology, writing – review and editing, project administration, resources.

## Ethics Statement

All procedures were conducted in accordance with the Purdue University Institutional Review Board.

## Consent

Written informed consent was obtained from the participants’ legal guardian or next of kin.

## Conflicts of Interest

M.L. contributed to the development of the ACLEW algorithm evaluated in this study (Lavechin et al. 2020; Räsänen et al. 2021). He has no financial interest in the ACLEW algorithm and does not receive monetary compensation for its use. While every effort has been made to maintain objectivity in the analysis and interpretation of results, this involvement is disclosed in the interest of full transparency.

## Supporting information




**Supporting File 1**: desc70239‐supp‐0001‐SuppMat.docx

## Data Availability

Anonymized transcripts and Python code to run the analyses are available at https://github.com/MarvinLvn/neurogen. Performance metrics reported in this paper are also available in tabular format at https://doi.org/10.17605/OSF.IO/5QE4M .

## Endnotes

1 To our knowledge, no performance difference across languages has been documented for LENA, even when the system was applied in a different linguistic and sociocultural setting (see Cristia et al. 2021 for an investigation of LENA's performance on Tsimane).

2 Segmentation + Conversation analysis: https://github.com/MarvinLvn/voice‐type‐classifier

3 Adult word count: https://github.com/orasanen/ALICE

4 Vocalization classification: https://github.com/LAAC‐LSCP/vcm
