# Supplementary material for: Performance and Biases of the LENA and ACLEW Algorithms in Analyzing Language Environments in Down, Fragile X, Angelman Syndromes, and Populations at Elevated Likelihood for Autism
Source: Dev Sci. 2026 Jul 8;29(5):e70239. doi: 10.1111/desc.70239 (PMC13343393; doi:10.1111/desc.70239)
Supplement: Supplementary file 1 — Supporting File 1: desc70239‐supp‐0001‐SuppMat.docx [file DESC-29-e70239-s001.docx]

# Supplementary Information

## Supplementary Figures

| 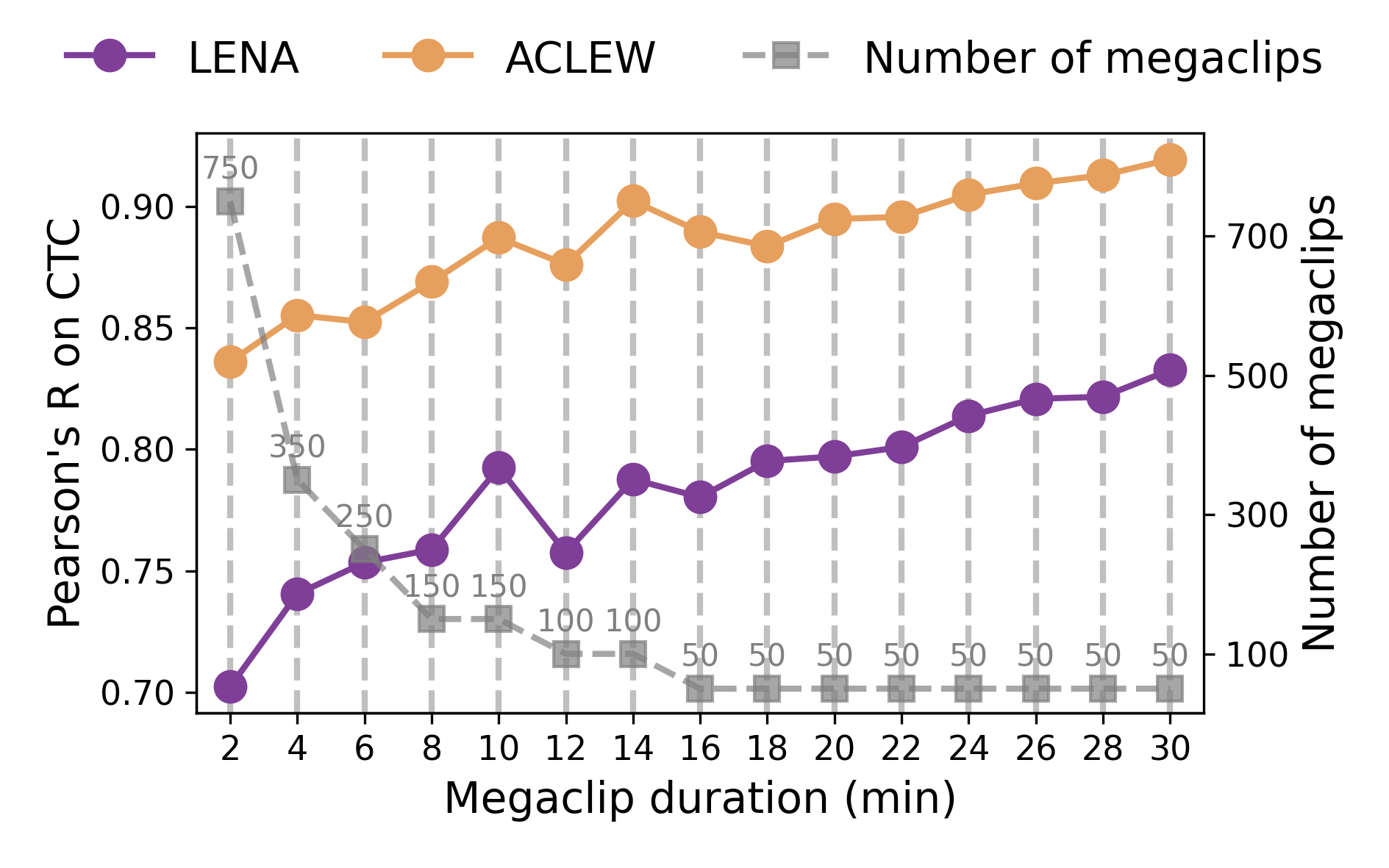 |
| --- |
| **Supplementary Figure 1**: Conversational Turn Count (CTC) Pearson’s *r* between human and automatic counts (LENA^®^ in purple and ACLEW in orange) as a function of megaclip duration. First, Pearson’s *r* are computed on all 2-min clips (750 clips) as in [Cristia et al. (2021)](https://www.zotero.org/google-docs/?UMH2Nd). We then combine successive 2-min clips (from the same daylong recording) into longer megaclips of 4-min (350 clips), 6-min (250 clips), etc.—discarding clips that cannot be combined. This graph shows how performance metrics on short clips underestimate performance over longer timescales. |
